# Food predictability and social status drive individual resource specializations in a territorial vulture

**DOI:** 10.1038/s41598-018-33564-y

**Published:** 2018-10-11

**Authors:** Thijs van Overveld, Marina García-Alfonso, Niels J. Dingemanse, Willem Bouten, Laura Gangoso, Manuel de la Riva, David Serrano, José A. Donázar

**Affiliations:** 1 Department of Conservation Biology, Estación Biológica de Doñana (CSIC), Américo Vespucio 26, E-41092 Sevilla, Spain; 2 Department of Biology, Ludwig-Maximilians-Universität München (LMU), Großhaderner Strasse 2, 82152 Planegg-Martinsried, Germany; 3 Theoretical and Computational Ecology, IBED, University of Amsterdam, PO Box 94248, 1090 GE Amsterdam, The Netherlands

## Abstract

Despite increasing work detailing the presence of foraging specializations across a range of taxa, limited attention so far has been given to the role of spatiotemporal variation in food predictability in shaping individual resource selection. Here, we studied the exploitation of human-provided carrion resources differing in predictability by Canarian Egyptian vultures (*Neophron percnopterus majorensis*). We focussed specifically on the role of individual characteristics and spatial constraints in shaping patterns of resource use. Using high-resolution GPS data obtained from 45 vultures tracked for 1 year, we show that individual vultures were repeatable in both their monthly use of predictable and semi-predicable resources (feeding station vs. farms) and monthly levels of mobility (home range size and flight activity). However, individual foraging activities were simultaneously characterized by a high degree of (temporal) plasticity in the use of the feeding station in specific months. Individual rank within dominance hierarchy revealed sex-dependent effects of social status on resource preference in breeding adults, illustrating the potential complex social mechanisms underpinning status-dependent resource use patterns. Our results show that predictable food at feeding stations may lead to broad-scale patterns of resource partitioning and affect both the foraging and social dynamics within local vulture populations.

## Introduction

Foraging tactics are often highly plastic, allowing individuals to adaptively respond to spatial and temporal fluctuations in resource availability and environmental changes. Yet, despite plasticity in foraging, individuals of the same species typically tend to use only a subset of the resources available in the environment, often displaying distinct foraging specializations and/or dietary preferences^1^. Such individual divergence in resource use may arise from phenotypic differences (e.g., morphology, behaviour, and physiology) and environmental constraints affecting foraging trade-offs^1^. As such, foraging specialisations are generally thought of as different foraging optimization strategies^2,3^. However, the extent to which the interplay between ecological conditions and individual resource preferences affects (or determine) population spatial dynamics remain poorly understood^4^.

A major determinant of individual foraging strategies is the spatial clumping and temporal predictability of resources, affecting decisions on patch choice, patch departure times and inter-patch movements^3^. Although temporal predictable food patches (pulsed resources) characterize most natural systems (e.g., upwellings, insect outbreaks, carcasses^5^), the presence of anthropogenic food resource pulses (e.g., fishing discards, refuse dumps, feeding stations) are increasingly impacting the natural dynamics of food supply, creating new ecological conditions upon which many animals base their foraging decisions^6^. Predictable food patches can greatly alter the costs and benefits of foraging, either by reducing the time and energy needed for food searching^7^ or by increasing levels of competition among individuals attracted to the same resource and hence, the costs associated with food acquisition^8,9^.

While high levels of competition and interference at predictable food may lead to the exclusion of subordinate individuals from these resources, the mechanisms underlying the usage of locally superabundant food are likely more complex. First, the ‘economic defendability’ of this resource is typically low^10^. While dominant individuals may be able to monopolize a large share of the food, there will always be some food available to lower-ranked individuals. For example, predictable food patches are often visited by individuals for which the costs of food searching may be high, such as young, inexperienced individuals or immigrants from other populations with limited knowledge of their environment^11^. Second, for territorial birds, central place foraging constraints during breeding may importantly affect the ability of making use of predictable food^12^. Such constraints may in turn differ among individuals depending on social rank if, for instance, the location of predictable food influences local territory quality^13,14^. Although there is increasing evidence that predictable anthropogenic resources are used by only a subset of the population, including seabirds^15–17^ and storks^11^, studies examining the potential trade-offs responsible for the asymmetric use of anthropogenic food are still scarce (but see^11^).

European vultures currently rely on feeding stations (where abundant food is supplied on a regular basis^18^) and, to a lower extent, on the surroundings of cattle farms (more places, but where food is scarcer and less predictable^19^). Despite concerns about the potential impact of such large feeding stations on the natural foraging habits and social structure of local vulture populations^20–22^, a mechanistic understanding of how the use of surplus food varies within and among individuals is still lacking. The extent to which changes in the spatial distribution and predictability of resources may influence behavioural processes key to understand spatial population dynamics therefore still remain poorly understood.

Here, we assessed individual plasticity and repeatability in the exploitation of food resources varying in predictability by a territorial, but highly social avian scavenger. We took advantage of a twenty-year research program on a closed island population of the endangered Canarian Egyptian vulture (*Neophron percnopterus majorensis*). Continuous monitoring has resulted in 90% of the birds (total population size c.a. 300 individuals) being ringed in 2016, 16% of them carrying GPS-transmitters (n = 45). Firstly, we described seasonal variation in the monthly use of food resources varying in their spatiotemporal predictability (i.e. highly predictable feeding stations and garbage dump, versus semi-predictable goat/sheep farms) and how vultures allocated their time into flight and non-flight behaviour. Next, we tested our main hypothesis that individual vultures differ in resource preferences as a result of trait-specific foraging optimization strategies. In a first step, we assessed whether individual vultures were repeatable in their preferences for food resources differing in predictability (farms vs. feeding stations) and levels of mobility (home range size and flight activity). Second, we specifically test whether birds preferring predictable food reduce their overall food searching activities (i.e., showing both smaller home-ranges and reduced flight activity) by analysing both within- and among-individual correlations between resource use and mobility. Finally, we examine whether predictable food is of higher value to dominant birds, owing to their superior competitive skills, by analysing the effects of individual rank within a dominance hierarchy, as well as sex, age, and territorial status. In addition, in territorial birds, we examine the role of spatial constraints in shaping individual resource use and patterns of mobility, by analysing how territory location (i.e. distance to predictable feeding sites) may affect resource use and ranging behaviour, again, in relation to individual traits.

## Results

### Temporal dynamics in resource use and movement pattern

**Figure 1:**
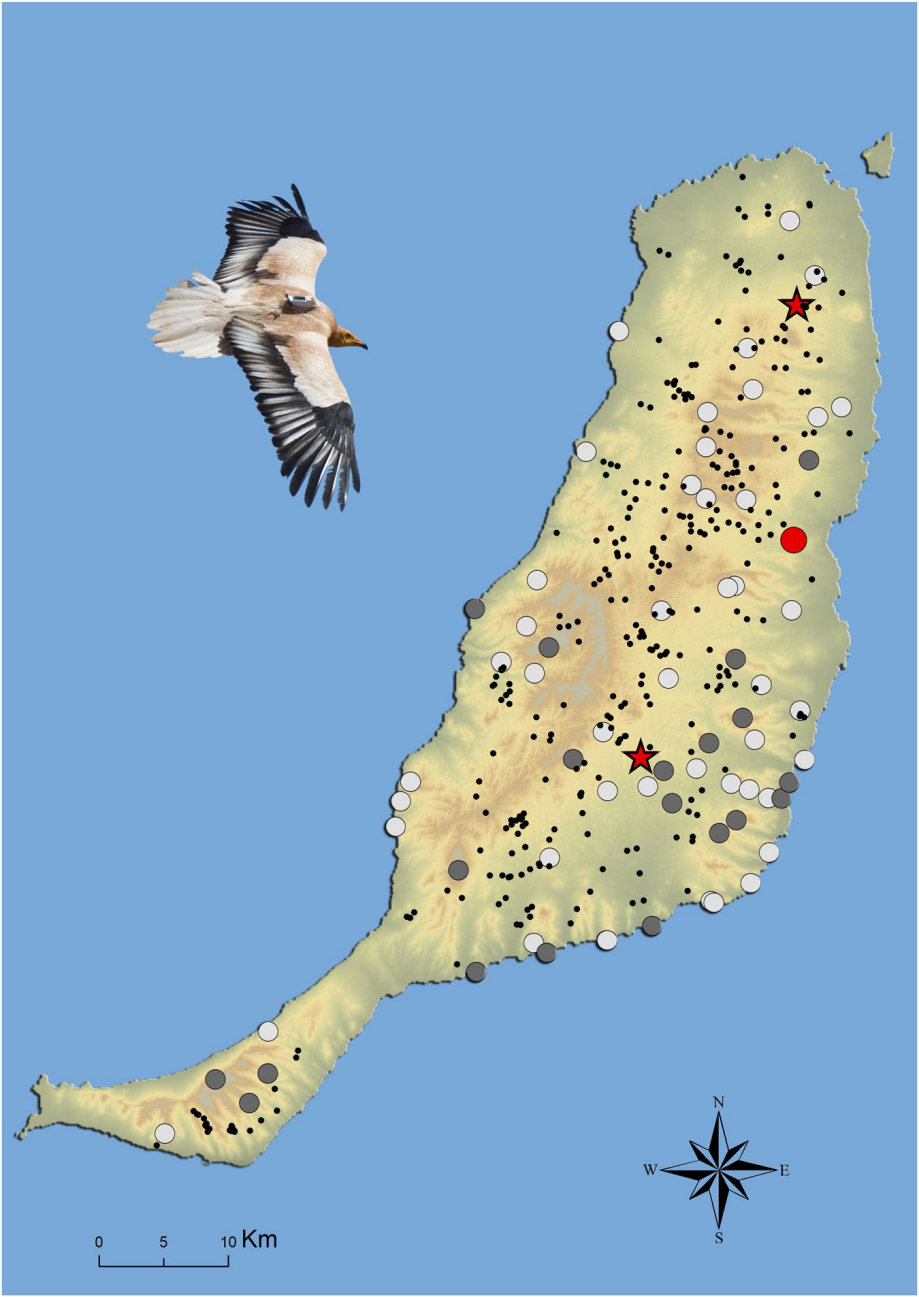
Overview of Fuerteventura showing the availability of semi-predictable resources (farms, black dots, n = 319), and predictable resources (two feeding stations at approximately 40 km distance (red stars) and garbage dump (red dot)). The grey dots represent the territories of GPS-logged territorial birds (11 females and 9 males) and the white dots all occupied territories in 2016 (n = 60).

The overall monthly proportion of time spent at human-provided food resources (feeding stations, farms, garbage dump; all pooled, see Fig. 1 for an overview) was 14.7% ± 9.8 SD. Vultures spent most time at the central feeding station and to a lesser extent at farms (average monthly proportion of time 8.5% ± 9.0 SD and 3.9% ± 4.6 SD, respectively). The garbage dump and the feeding station located in the north, were rarely used (average 1.5% ± 2.4 SD and 0.9% ± 2.3 SD, respectively). Monthly time budgets varied strongly over the course of the season. As a general pattern, average monthly flight activity was generally low during the non-breeding season (July–December) and substantially increased during the breeding season (January–June). By contrast, average monthly time spent at the central feeding station, and to a lesser extent also at farms, decreased during the breeding season and again increased during the non-breeding season (Fig. S2, details in Text S2 and below).

### Individual repeatability and plasticity in resource use and mobility

There was significant among-individual variance in the intercepts (i.e. non-adjusted individual repeatability) of the time spent at the central feeding station (variance ± SE = 0.43 ± 0.10, χ^2^_1_ = 175.6, p < 0.001), farms (0.45 ± 0.11, χ^2^_1_ = 203.4, p < 0.001) and the ratio feeding station: farms (‘resource preference’: 0.47 ± 0.11, χ^2^_1_ = 209.1, p < 0.001). Both measures of mobility were also individually repeatable: logKDE95 (0.43 ± 0.10, χ^2^_1_ = 184.8, p < 0.001), and flight activity (0.18 ± 0.06, χ^2^_1_ = 39.8, p < 0.0001).

**Figure 2:**
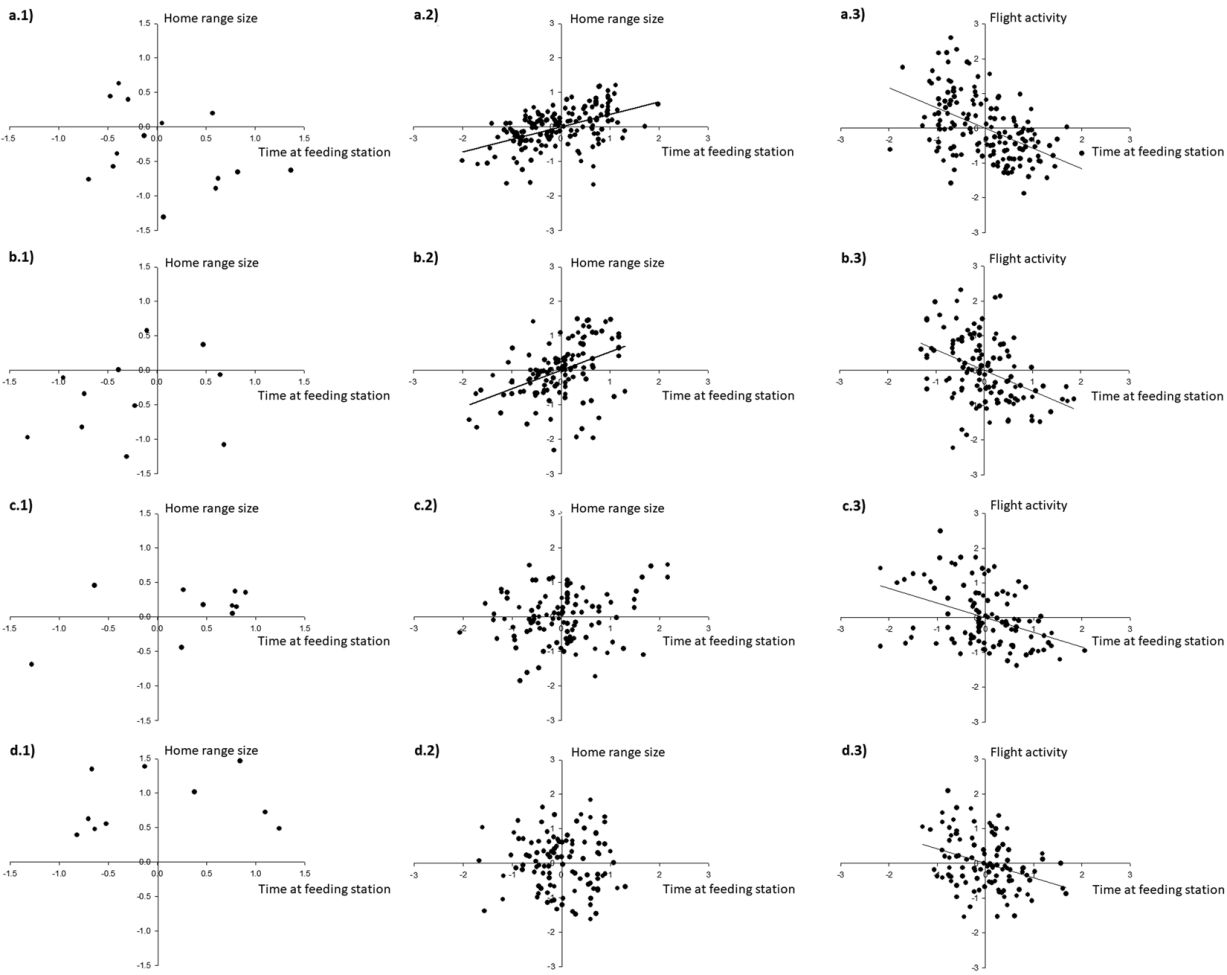
Within- and among-individual correlations between mobility parameters (home range size and flight activity) and time spent at the central feeding station in Egyptian vultures for (**a**) territorial females, (**b**) territorial males, (**c**) non-territorial females and, (**d**) non-territorial males, tracked between October 2015 and September 2016. (a.1–d.1) among-individual correlations (n = 45 individual means), (a.2–d.2,a.3–d.3) within-individual correlation (n = 486 months). The plot of the among-individual correlation is visualized as the correlation between means of each individual trait; the within-individual correlation is visualized as the correlation between the deviations of each monthly observation from a focal individual’s mean for each trait.

There was no significant within-individual or among-individual covariance between the use of farms and feeding stations (Table 1), nor among-individual covariance between resource use and mobility parameters (Table 1, for territorial and non-territorial males and females, Fig. 2a1,d1). Positive within-individual covariance was found between the use of the feeding station and LogKDE95 (χ = 34.4, p < 0.0001, Table 1), while negative within-individual covariance was found between time spent at the central feeding station and flight activity (χ = 89.1, p < 0.001, Table 1), indicating that individual birds increased home-ranges and decreased flight activity in those months when they visited the feeding station more frequently. A similar relationship was found for resource preference (χ = 27.1, p < 0.001 and χ = 20.6, p < 0.001, for covariance with flight activity and LogKDE95, respectively), but not the use of farms, indicating that the effects of resource preference on mobility were driven by the use of the feeding station (Table 1). Within-individual covariance between time spent at the central feeding station and mobility parameters (home-range size and flight activity) were both significant in territorial birds (females: 0.23 ± 0.04, χ = 39.3, p < 0.001 (Fig. 2a.2,a3) and −0.37 ± 0.07, χ = 39.1, p < 0.001; males: 0.25 ± 0.06, χ = 24.2, p < 0.001 and −0.26 ± 0.06, χ = 21.6, p < 0.001 (Fig. 2b.2,b.3)), while for non-territorial females and males, significant within-individual covariance was found only between time spent at the central feeding station and flight activity (p < 0.001, non-territorial females: Fig. 2c.2,c.3, non-territorial males: Fig. 2d.2,d.3).

**Table 1:** Estimated within- and among-individual covariances and correlations between mobility parameters (home range size (logKDE95), and flight activity (time spent flying)), resource use (time spent at farms and at the central feeding station, square-root transformed), and resource preference (ratio time spent at the feeding station:farms, arctangent transformed) of Egyptian vultures on Fuerteventura extracted from a bivariate mixed model with no covariates.

	Activity		Home range		Farms		Feeding station	
	Covariance ± S.E.	r	Covariance ± S.E.	r	Covariance ± S.E.	r	Covariance ± S.E.	r
(a) Within-individual correlation								
Home range	0.03 ± 0.03	0.10						
Farms	0.05 ± 0.03	−0.10	0.04 ± 0.03	0.07				
Feeding station	**−0**.**30** ± **0**.**04**	**−0**.**42**	**0**.**15** ± **0**.**03**	**0**.**27**	0.01 ± 0.03	0.01		
Resource preference	**−0**.**16** ± **0**.**03**	**−0**.**24**	**0**.**11** ± **0**.**03**	**0**.**21**	**−0**.**20** ± **0**.**03**	−0.37	**0**.**39** ± **0**.**10**	0.77
(b) Between-individual correlation								
Home range	0.03 ± 0.03	0.08						
Farms	**−**0.02 ± 0.05	**−**0.05	**−**0.04 ± 0.07	**−**0.05				
Feeding station	0.01 ± 0.05	0.02	0.04 ± 0.07	0.09	−0.10 ± 0.07	−0.23		
Resource preference	0.02 ± 0.05	0.05	0.06 ± 0.07	0.14	**−0**.**27** ± **0**.**09**	**−**0.60	**0**.**42** ± **0**.**03**	0.87

Significant correlations (p < 0.05) are printed in bold.

### Effects of social status on resource use and mobility

**Figure 3:**
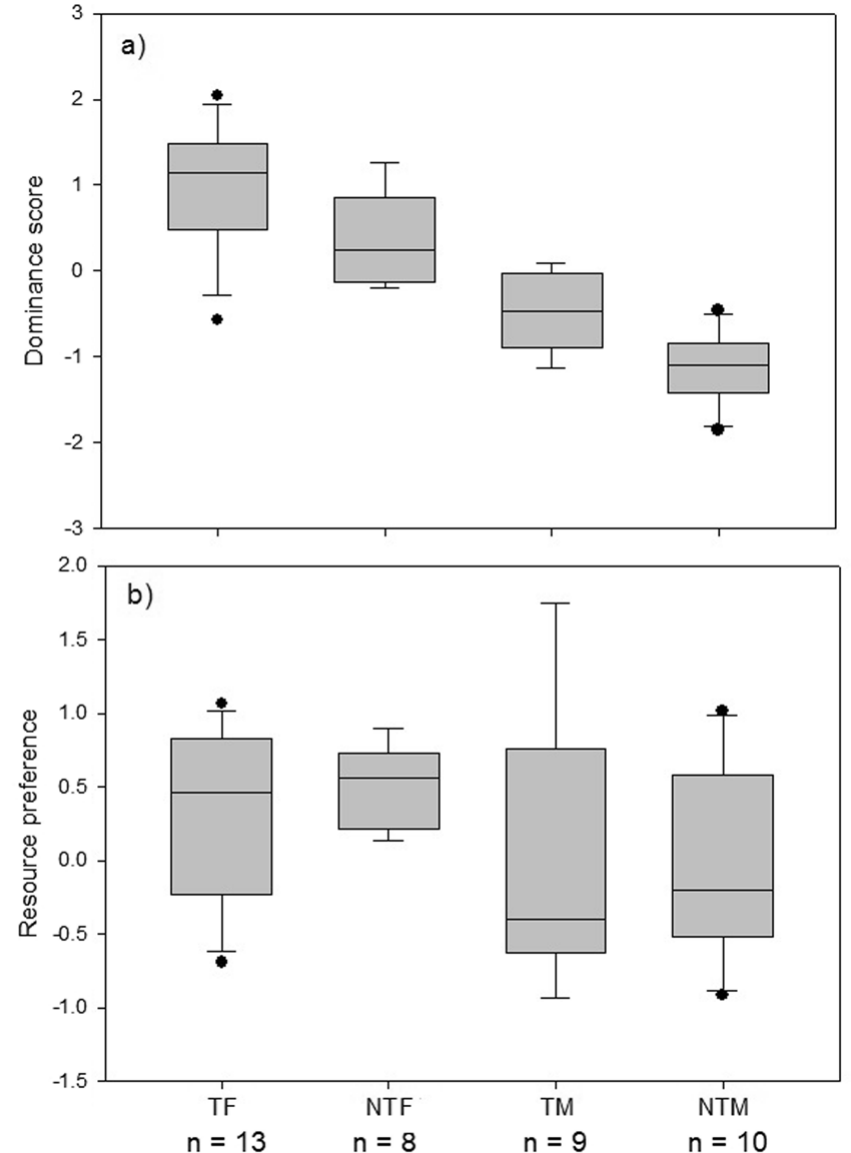
Differences in (**a**) dominance rank (high values indicating high ranks) and (**b**) resource preference (ratio time spent at feeding stations:time spent at farms (scaled by the standard deviation and mean-centred) for 40 Egyptian vultures fitted with GPS loggers categorized according to sex and territorial status (TF: territorial females, NTF: non-territorial females, TM: territorial males, NTM: non-territorial males).

Females were socially dominant over males, while within sexes, territorial birds were socially dominant over non-territorial birds (territorial females > non-territorial females > territorial males > non-territorial males, Fig. 3a). Social status also increased with age (Spearman rank correlation r = 0.52, p < 0.001, n = 40).

#### Resource specialization

Resource preference differed between the sexes in relation to dominance rank and territorial status (Table 2), and these effects varied strongly across the season (month squared × sex × dominance rank: *F*_3,387_ = 8.38, p < 0.001; month squared × sex × territorial status: *F*_2,387_ = 6.80, p = 0.001). Resource preference also varied according to age, with young birds spending more time at the central feeding station than farms compared to older birds (Table 2). Overall, females preferred the central feeding station over farms, while males showed the opposite pattern (Fig. 3b). The importance of individual traits in explaining resource preference is further illustrated by a decrease in among-individual variance changing individual repeatability (non-adjusted: R = 0.47 vs. adjusted: R = 0.28, Table 2).

**Table 2:** Results of GLMM on seasonal effects and individual attributes affecting monthly resource preference (ratio time spent at the feeding station:farms, arctangent transformed), home range size (logKDE95) and flight activity (time spent flying, square-root transformed) of 45 Egyptian vultures tracked with GPS-loggers on Fuerteventura (Spain).

Fixed effects	a) Resource preference			a) Home range size		c) Activity			
	β (SE)	F_NUMdf, DENdf_	*P*	β (SE)	F_NUMdf, DENdf_	*P*	β (SE)	F_NUMdf, DENdf_	*P*
Intercept	**−**0.52 (0.23)		0.0308	**−**0.73 (0.25)		0.0053	1.31 (0.23)		<0.0001
Sex	−0.28 (0.33)	2.65_1,58.2_	0.11	0.25 (0.36)	0.95_1,55.7_	0.33	**−**0.96 (0.33)	4.94_1,51.9_	**0**.**0306**
Age	−0.27 (0.09)	9.33_1,32_	**0**.**0045**	0.05 (0.10)	0.27_1,32.8_	0.61	0.10 (0.09)	1.17_1,31.5_	0.29
Territorial status	−0.80(0.33)	0.02_1,51.4_	0.88	2.21 (0.35)	46.67_1,49.6_	** <0**.**0001**	−0.06 (0.33)	0.40_1,46.4_	0.53
Dominance rank	−0.68 (0.31)	0.19_1, 52.8_	0.67	−0.03 (0.33)	0.04_1,50.8_	0.84	0.47 (0.33)	4.22_1,47.5_	**0**.**0456**
Month	0.08 (0.03)	6.29_1, 393_	**0**.**0125**	−0.12 (0.03)	12.67_1,392_	**0**.**0004**	0.12 (0.03)	14.80_1, 391_	**0**.**0001**
Sex*dominance rank	1.53 (0.37)	17.48_1, 53.9_	**0**.**0001**	0.01 (0.34)	0.00_1, 51.8_	0.95	−0.15 (0.37)	0.17_1, 48.4_	0.68
Sex*territorial status		15.55_1, 53.9_	**0**.**0002**		7.08_1, 51.7_	**0**.**0103**		0.84_1, 48.4_	0.36
Month²	0.17 (0.11)	15.34_1, 389_	**0**.**0001**	0.23 (0.11)	1.69_1, 389_	0.19	−1.02 (0.10)	126.7_1, 388_	**<0**.**0001**
Month²* sex*dominance rank		6.80_2, 387_	**0**.**0012**		4.15_2, 387_	**0**.**0165**		4.28_2, 386_	**0**.**0145**
Month²* sex*territorial status		8.38_3, 387_	**<0**.**0001**		25.14_3, 387_	**<0**.**0001**		3.19_3, 386_	**0**.**0236**
**Random effects**	**σ² (SE)**	**Z**	**P**	**σ² (SE)**	**Z**	**P**	**σ² (SE)**	**Z**	**P**
Individual	0.18 (0.06)	3.20	0.0007	0.22 (0.07)	3.35	0.0004	0.21 (0.06)	3.33	0.0004
Residual	0.46 (0.03)	13.81	**<**0.0001	0.47 (0.03)	13.82	**<**0.0001	0.40 (0.03)	13.79	**<**0.0001
**Adjusted repeatability**	***R***	**χ²**	**P**	***R***	**χ²**	**P**	***R***	**χ²**	**P**
	0.28	64.7	**<**0.0001	0.32	82.3	**<**0.0001	0.34	85.8	**<**0.0001
**Null-model**	**σ² (SE)**	**Z**	**P**	**σ² (SE)**	**Z**	**P**	**σ² (SE)**	**Z**	**P**
Individual	0.47 (0.11)	4.23	**<**0.0001	0.43 (0.10)	4.18	**<**0.0001	0.18 (0.06)	3.20	0.0007
Residual	0.53 (0.04)	14.86	**<**0.0001	0.56 (0.04)	14.87	**<**0.0001	0.83 (0.06)	14.85	**<**0.0001
**Non-adjusted repeatability**	***R***	**χ²**	**P**	***R***	**χ²**	**P**	***R***	**χ²**	**P**
	0.47	209.1	**<**0.0001	0.43	184.8	**<**0.0001	0.18	39.8	**<**0.0001

All transformed response variables were scaled and centred. Repeatability (R) was calculated as the among-individual variance divided by the sum of the among-individual and the residual (within-individual) variance, and its significance tested by comparing models with and without the random effect of bird ID using a likelihood ratio test.

**Figure 4:**
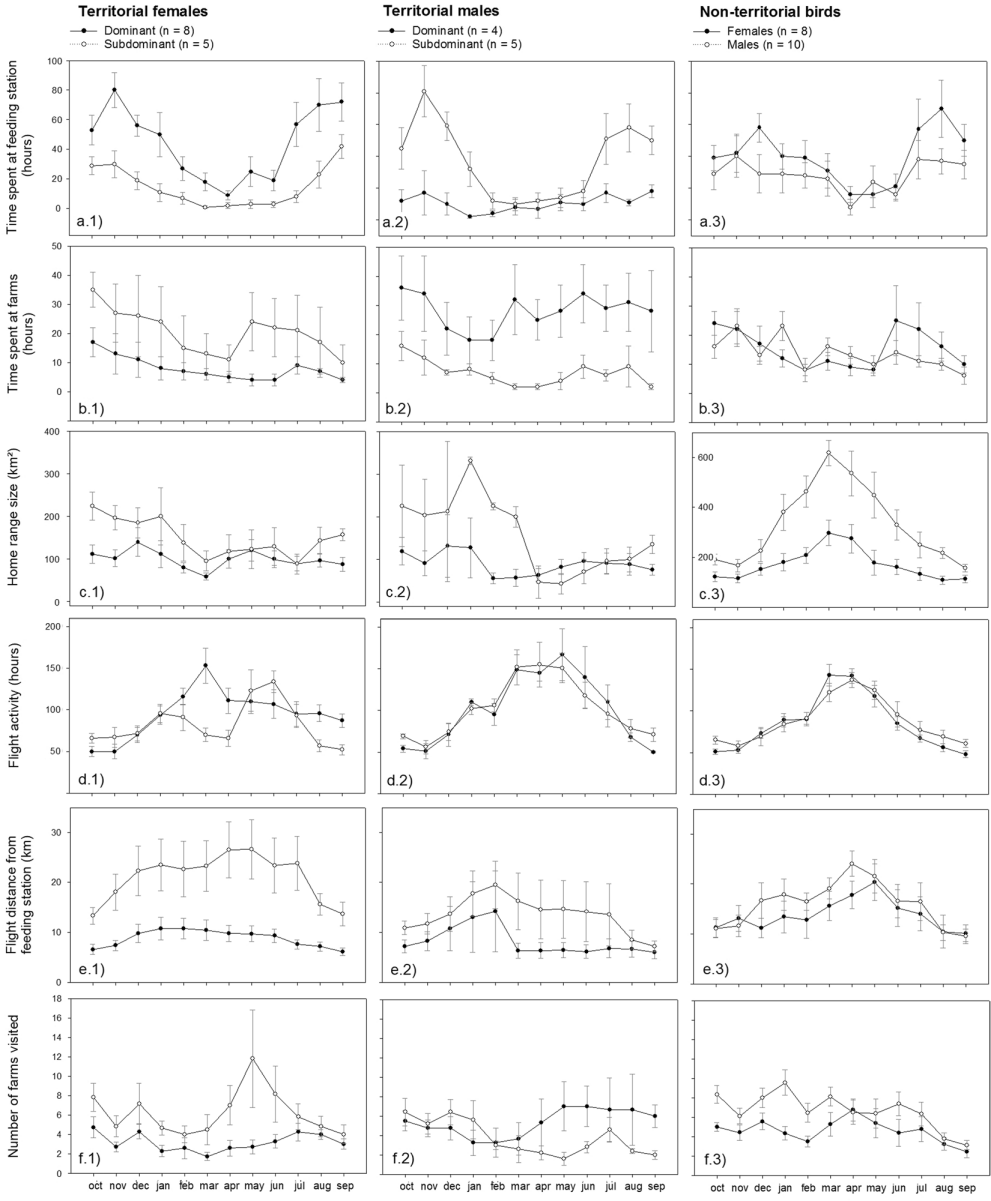
Overview of monthly variation in (**a**) time spent at the central feeding station and (**b**) farms (all pooled), (**c**) home range size (95% Kernel Density Estimate (KDE)), (**d**) flight activity (number of hours flying), (**e**) flight distance from the central feeding station, and (**f**) the total number of farms visited. Data for territorial and non-territorial male and female Egyptian vultures tracked with GPS-loggers on Fuerteventura between October 2015 and September 2016. For illustrative purposes, territorial males and females were categorized according to dominance status (dominant above and subdominant below the median), but included as a continuous variable in statistical models. In non-territorial males and females no effects of dominance was present. Note that the breeding activities of vultures occur between January and July (with the peak in egg-laying occurring in March).

However, within territorial birds, a reversed effect of social status on resource use was found in males and females (sex × dominance rank *F*_1,18_ = 9.6, *β* = 1.58 ± 0.52, p = 0.006), showing a positive and negative correlation respectively (females: Fig. 4a.1 and a.2; males: Fig. 4b.1,b.2). Within non-territorial males and females, no correlation was found between dominance rank and time spent at either the central feeding station or farms (p > 0.11), nor were there significant sex-difference in time spent at different resources (Fig. 4a.3,b.3). Subdominant territorial females visited more farms (p = 0.02), which showed a clear peak in use during the late chick-rearing phase (Apr-May) (p = 0.01, Fig. 4f.1). In male territorial birds there was no relationship between dominance rank and number of farms visited (p = 0.15, Fig. 4f.2). Details on statistics are provided in Table S1.

#### Mobility

Links between social status and mobility varied according to sex and territorial status, but these patterns were highly seasonal and/or only present during specific periods of the year (Table 2). Repeatability indices for home-range size and flight activity changed due to respectively a decrease in among-individual variance component (non-adjusted: R = 0.43 vs. adjusted: R = 0.32, Table 2) and increase in within-individual variance component (non-adjusted: R = 0.18 vs. adjusted: R = 0.34, Table 2), indicating effects of individual traits on the ranging behaviour and confounding seasonal effects on flight activity.

Subdominant territorial males and females had larger home ranges during late summer (Oct–Dec) and the pre-egg laying phase (Jan-Mar) compared to dominant individuals, but these differences disappeared during the remaining part of the year (month squared × dominance rank; females: p =< 0.001, Fig. 4c.1; males: p = 0.052, Fig. 4c.2). In both territorial males and females, flight activity peaked during the breeding season (p < 0.001 for both sexes; females: Fig. 4d.1; males: Fig. 4d.2). Non-territorial birds, but males in particular, made large scale movements during the breeding season (p = 0.001, Fig. 4c.3), corresponding to a peak in flight activity and large flight distance relative to the feeding station around the egg-laying phase (Mar-Apr) of breeding birds (p < 0.001 for both sexes, Fig. 4d3,e3). Overall, non-territorial males visited more farms than non-territorial females (p = 0.012, Fig. 4f.3). Details on statistics are provided in Table S1.

#### Territory location and use of the feeding station

**Figure 5:**
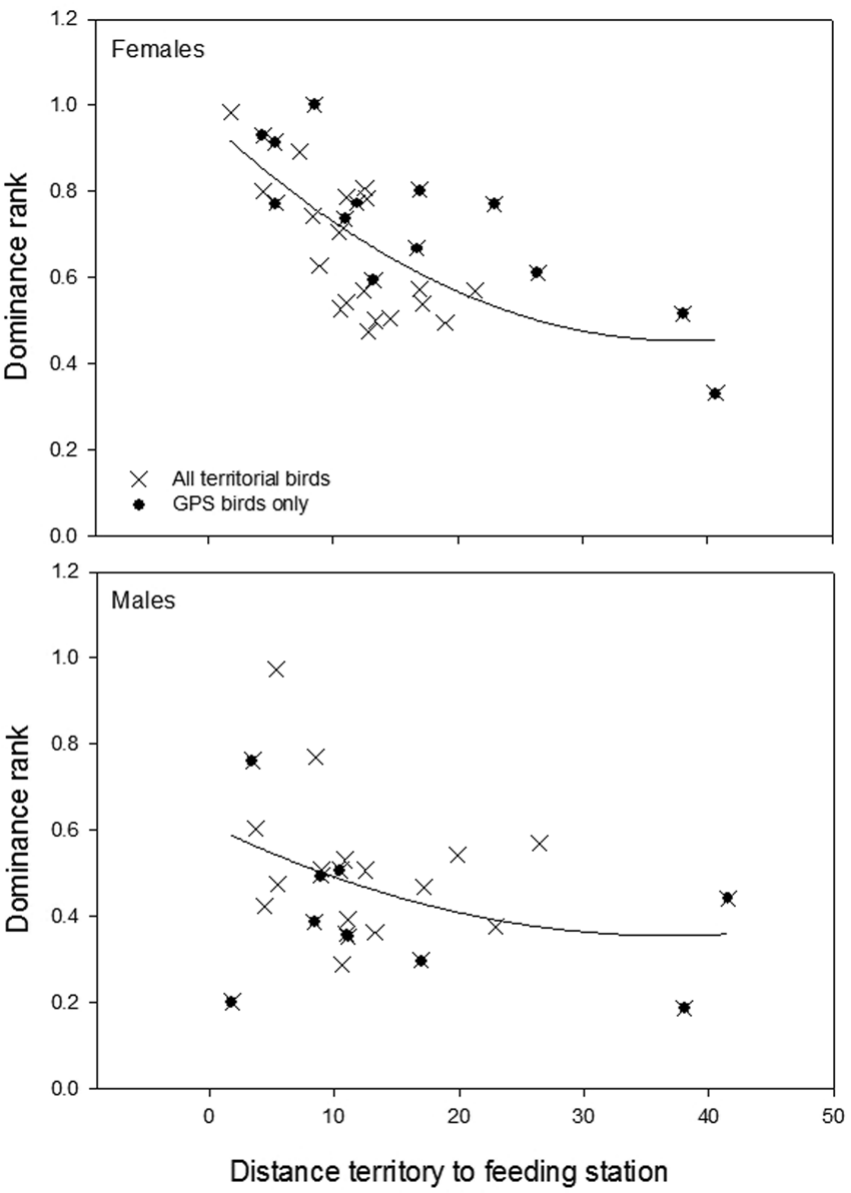
Relationship between dominance rank and distance from the breeding territory to the central feeding station (km) for male and female Egyptian vultures on Fuerteventura in 2016. Black dots represent birds fitted with GPS-loggers (13 females and 9 males) and crosses birds with known dominance rank (36 females and 25 males). The relationship in females is significant (Spearman rank correlation, r = −0.77, p = 0.002, and r = −0.59, p = 0.0001, respectively), while non-significant in males (p = 0. 55 and p = 0.18 respectively).

High-ranked females bred closer to the feeding station (Spearman rank correlation, r = −0.77, p = 0.002, n = 13, including birds without GPS loggers r = −0.59, p = 0.0001, n = 36, Fig. 5a), with distance from the territory to the feeding station being negatively correlated with time spent at the central feeding station (*F*_1,18.1_ = 11.10, *β* = 0.60 ± 0.18, p = 0.004). In males, there was no relationship between dominance rank and distance between territory and the central feeding station (Spearman rank correlation, GPS-birds: r = −0.23, p = 0.55, n = 9; including birds without GPS-logger: r = −0.28, p = 0.18, n = 25, Fig. 5b). Distance from the territory to the feeding station also tended to be negatively correlated with time spent at the feeding station (p = 0.06). Overall, flight distances relative to the feeding station were much larger in subdominant territorial females (p < 0.001), except during summer months (month squared × dominance rank: p < 0.001, Fig. 4e.1). In males, social status was unrelated to flight distance to the central feeding station (p = 0.31, Fig. 4e.2).

## Discussion

We found strong evidence for level-specific associations between individual resource use and mobility, providing novel insights into the use of human-provided carrion by social avian scavengers. Our detailed quantification of individual rank order differences revealed different effects of social status on resource preference in breeding males and females, illustrating the potential complex social mechanisms underpinning individual resource use patterns.

### Resource preferences and individual movement behaviour

While feeding stations may have a profound impact on the foraging dynamics of local vulture populations^23^, studies investigating individual-level responses to these feeding practices are almost non-existent^12,22^. We found that competitive superior individuals (females, the larger sex in this and most raptor species) made consistent use of the feeding station throughout the year. By contrast, subdominant individuals (males in particular) favoured farms as their main source of food, most likely because of reduced competition at these sites due to the unpredictable nature of food supply. These results demonstrate for the first time the asymmetric use of this widely used conservation tool in vultures, and point towards a major role of feeding stations in driving patterns of resource partitioning within populations.

Importantly, vultures preferring farms did not necessarily have larger home-ranges compared to birds feeding mostly at the central feeding station, despite both traits being individually repeatable. By contrast, ranging behaviour and flight activity varied plastically within individuals as a function of the use of the central feeding station. This shows that all individuals may temporally increase their use of predictable food, indicating that the feeding station may serve an important function as a food insurance (see also^24^). Patterns of within-individual plasticity between resource use and ranging behaviour were most pronounced in birds breeding far away from the feeding station (mostly foraging at farms) making large-scale flights to the central feeding station. Thus, instead of searching for unpredictable carcasses at farms (or over wider natural areas), these vultures may frequently leave their habitually exploited home range to visit the central feeding station (i.e. sometimes even including birds breeding on Lanzarote^12^). The temporal nature of these movements may be explained by birds facing various trade-offs: they may choose to forage at predictable feeding site in order to reduce food searching efforts, but face potentially higher levels of competition for food at this site compared to farms, while the energetic costs associated with large-scale movements to this site may be substantial. Indeed, the movement behaviour of vultures on Fuerteventura may be importantly shaped by “alisios” winds, the dominant northeast trade winds (with an average speed of 20 km/h) that are particularly strong during spring and summer^25^. In addition, time-displacement constraints may be particularly important for single-prey loaders such as Egyptian vultures, notably during chick rearing (May–July). Overall, these patterns point towards differences in foraging costs associated with territory distance to the feeding station, which at least in females seems associated with social status (see below).

The important role of spatial constraints, and central-place foraging task in particular, in shaping resource use patterns is further illustrated by the strong seasonal plasticity in the use of the feeding station. Outside the breeding season, all tracked individuals, except dominant and non-territorial males, spent more time at the central feeding station and hence, decreased their flight distance relative to this site. Large-scale and straight-line movements to predictable feeding sites seem to be general among Egyptian vultures, and has been previously observed in mainland Spain^26^. However, as we showed here, these movements may be part of a much more complex foraging and spatial dynamics, highly influenced by the existence of a single, predictable feeding station.

### Social mechanism underlying individual resource preference

While it is recognized that social status shapes individual foraging decisions, we are currently not aware of other studies showing the existence of consistent, year-round differences in status-dependent resource specialisation in relation to resource distribution and predictability. Furthermore, although dominance status plays an important role in determining carcass and predictable resource exploitation in obligate scavengers^27–30^, sex- and individual-rank order differences in resource use have been very rarely quantified in vultures^31^. As such, a key finding of our study is that effects of social rank on resource use were reversed within territorial males and females, thus, showing that rank-specific foraging trade-offs can be complex, and shape resource preferences differently within each sex.

In territorial females, resource preferences depended on the distance of the territory to the feeding station, with dominant birds breeding at closer distances and spending more time at this resource, and low-ranked birds breeding further away and, consequently, relying more on farms. This suggests that the location of territories with respect to predictable food may be the main mechanism shaping status-dependent resource use in this sex. More specifically, securing a territory close to the feeding station may be beneficial in terms of knowledge on the occurrence of food dumps, and reduced time-displacement costs during chick rearing, while at the same time, dominant birds have instant access to food provided at this site since they are able to push most other birds away from food. In contrast, high-ranked territorial males spent consistently more time at farms throughout the year, while low-ranked territorial males spent little time at both farms and feeding station while breeding, suggesting they may generally rely more on natural food resources (see also^32^). Outside the breeding season, the absence of central place foraging tasks, as well as the probable lower availability of food, may drive low-ranked males to exploit predictable food at the central feeding stations, despite a high cost-benefit ratio of resource acquisition due to their low social position. As opposed to females, the despotic distribution of territories in males may be determined by the presence of high quality food dumps near farms (i.e., frequent supply of livestock) instead of distance to the feeding station. However, future studies should unravel the differential cost associated with foraging for natural vs. semi-unpredicatble resources and the role of farms in determing territory quality to fully understand rank-specific foraging trade-offs in males.

A complementary scenario may be that subdominant territorial males can specifically visit the feeding station to improve their social rank and/or to search for new partners (as has been hypothesized in social corvids^33,34^). In fact, after breeding, large gatherings at the feeding station sometimes reaching up to 100 individuals, suggests that this site may also serve a function as a social meeting place (van Overveld *et al*. in prep).

Furthermore, competitive asymmetries among individuals may not be the only individual factor explaining differences in resource use. First, the observed sex-specific resource preferences may also be linked to asymmetries in foraging-roles between males and females, which is common in sex-dimorphic birds of prey^35^ and avian scavengers^31^. Consequently, sex-asymmetries in resource preference may not be the result of resource competition per se, but rather the result of a more general difference in foraging niche partitioning^36^, in which the sexes perceive the value of resources differently depending on levels of competition. Second, although non-territorial, adult males visited more farms (compared to females), this difference may have resulted from the large-scale explorative movements made by these males during the breeding season. These movements, peaking around the egg-laying phase, seem to be aimed at collecting information about potential future recruitment sites^37^ more than being the result of social competition. Finally, younger birds preferred the feeding station over farms which, given the overall low social position of young birds, suggests that this preference for a more competitive environment seems better explained by their poor explorative skills and limited environmental knowledge^11^. Probably, this age-effect also accounts for the high variance observed in resource preference in non-territorial males.

### Ecological and applied implications of surplus food

Predictable feeding sites may attract a large number of birds typically consisting of a mixture of individuals facing different foraging trade-offs, including permanent (dominant and non-territorial females, young inexperienced birds), and temporal visitors (subdominant females breeding far away from these feeding sites and males). Apart from individual traits explaining resource use patterns, our findings point towards a major role of spatial constraints in shaping the use of predictable food resources. Sex-differences in competitive abilities and/or foraging roles may create scenarios whereby males and females may perceive the value of territories differently regarding the location of predictable food, leading to complex patterns of resource partitioning and specialization. Overall, these results show that feeding stations may have a substantial impact on the social dynamics of local vulture populations, an issue that has so far received limited attention^18^. Future work, preferably by using food manipulation experiments, should unravel the exact influence of predictable food on sex-specific settlement patterns.

Future analyses should reveal the extent to which the differential use of predictable food may influence survival rates and reproductive output, and may change the selective pressures operating within populations. Strong negative effects on fitness may potentially arise if feeding stations are supplied with carcasses from intensive livestock farms rich in veterinary drugs^38^ or conversely, when feeding stations are situated in highly poisoned areas and are intended to serve as a poison-free place. For animals with a strong male- or female-based social structure, as is the case in many vulture species, feeding stations have the potential for driving asymmetric patterns of individual and/or sex-specific mortality within populations (see also^39^).

Lastly, the strong decrease in the use of the central feeding station during the breeding season (by dominant territorial and non-territorial females) suggest that vultures may switch to alternative carrion resource to feed their young and/or may generally include more natural carrion resources in their diet in periods of high abundance of such prey items^23^. More detailed studies are needed to deepen out the effectiveness of surplus food in relation to the dietary breath of different vulture species, which may help to further fine-tune conservation efforts.

To summarize, our results show that the food predictability and distribution may be an important driver underlying resource specialisation in vultures, whereby competition for predictable surplus food may drive individual resource preferences. In this way, predictable food may importantly affect both the foraging and social dynamics of local vulture populations. However, our results also evidence that other poorly known sex-specific mechanisms are at play, opening new research avenues. Overall, our study highlights the need to take into account social rank differences when studying patterns of individual resource specialization in highly social species.

## Materials and Methods

### Study species and population

The Canarian Egyptian vulture is a sedentary and endemic long-lived scavenger occupying the eastern part of the Canarian Archipelago (between 27.62°–29.42°N, and 13.33°–18.17°W). The species was once abundant throughout the archipelago^40^, but is currently classified as ‘critically endangered’ owing to severe population declines since the 20^th^ century. Fuerteventura Island (1662 km²) is the stronghold of the population, which is home to 60 breeding pairs and has an estimated population size of about 300 individuals in 2016 (authors unpublished). Extensive fieldwork (ringing of nestling and trapping of adults) has resulted in over 90% of the individuals being individually marked in 2016.

Egyptian vultures are facultative scavengers that forage solitary (or sometimes in couples). The species leads a vagrant but very social lifestyle prior to recruitment, but afterwards adults are much more solitary and territorial. Throughout the year, they may form large aggregations at places with an abundance of food and roosting often occurs communally^41^. On Fuerteventura, the species heavily relies on human-provided carcases available at goat and sheep farms throughout the island (Fig. 1, further details see below). At these farms, livestock carcasses appear irregularly in time. Slaughterhouse remains (pork heads and intestines, ±200 kg per week) are provided once or twice a week at each of the two feeding stations, one being located in the centre of the island (created in 1998) and one in the north (created in 2008) (Fig. 1). Local farmers regularly add additional livestock carcasses to both feeding stations. The feeding stations are separated by approximately 40 km distance. Vultures sometimes also forage at the garbage dump near the capital city Puerto del Rosario. Apart from these human-provided food resources, vultures may consume randomly encountered carcasses, especially those of feral goats and small-sized vertebrates such as wild rabbits (*Oryctolagus cuniculus*) and feral pigeons (*Columba livia*)^42^.

### GPS tracking

Vultures were captured with cannon-nets about 3 km away from the main feeding station in the centre of the island. GPS trackers (UvA-BITS, www.uva-bits.nl, University of Amsterdam, n = 26; e-obs, e-obs Digital Telemetry, Grünwald, Germany, n = 19) were attached to the bird using backpack harnesses. The total weight of the system varied between 31 g (UvABiTS) and 54 g (e-obs) (1.4–2.4% of the weight of the bird) which is assumed to be harmless to the individual^43^. In total, we used GPS-tracking data from 45 birds (24 females (14 territorial and 10 non-territorial) and 21 males (11 territorial and 10 non-territorial) collected over a 12-month period (October 2015– September 2016). GPS loggers were programmed to record locations every 1 to 5 minutes, but data were re-sampled to an interval of 10 minutes (range 9–11 minutes, R-function developed by D.S. Viana) to allow direct comparisons between individuals. Because of low-battery levels and/or poor satellite reception, intervals exceeding15 minutes were removed from the dataset. We only used GPS-fixes between sunrise and sunset. We included all months with continuous recording of GPS-locations, independent of downloading error (which was small due to sunny weather conditions). However, we excluded months for which we had incomplete logging data (e.g. due to exceedance of data logging capacity and mortality (N = 2). We also excluded all movements made to the neighbouring Lanzarote island (e.g. one bird breeds on Lanzarote, but spent its time outside the breeding season on Fuerteventura). In total, we had data available for 486 individual-month (994.179 fixes), comprising 12 months for 35 individuals and between 3 to 11 months for 10 individuals. The median percentage of time explained per month by GPS fixes was 94.8% (range 16.13–100%, further details below).

### Monthly activity budgets

To construct monthly activity budgets we distinguished between time allocated to flight vs. non-flight behaviour (including resting and foraging) using a threshold ground speed 3 m.s^−1^ for data obtained from both GPS devices (see Fig S1). Non-flight behaviour was further subdivided into time allocated to foraging at two types of food resources: highly predictable places (the two feeding stations and the garbage dump) and semi-predictable places (goat-sheep farms). To classify GPS data into resource use, we used the number of GPS locations within a buffer zone of 75 m around the centre of the two feeding stations and a buffer zone of 250 m around the centre of the garbage dump, covering respectively the total fenced area and the total surface of the garbage dump. Since farmers drop carcasses at variable distances from their farms, we used a buffer zone of 250 m to determine the use of farms as source of food (i.e. based on 10 farms where we knew the exact distance (median 254 m, range 60–610 m, García Alfonso *et al*. under review). In total, we were able to retrieve the coordinates of 319 out of 437 farms (73%). Farms differed greatly in size (median 202 animals, range 10–4217, n = 292). Our data included all farms with more than 500 animals (N = 67). Annual mortality rate of sheep and goats (including lambs) at farms is estimated at about 10% (see^44^). We described monthly variation in average time-budgets of all individuals (flight vs. non-flight behaviour), specifically detailing the time spent at resources differing in predictability in the non-flight category (the two feeding stations, garbage dump and all farms pooled). See Fig. S2 for an overview of average monthly time-budgets.

### Ranging behaviour

Monthly ranging behaviour per individual was quantified by calculating utilization distributions (UD) using the fixed kernel contour method adehabitHR package, R version 3.0.3^45^. Since we were interested in food searching behaviour and/or explorative movements, ranging behaviour was defined using the 95% kernel density estimate (95KDE, in km²). We excluded all non-flight GPS fixes at the feeding stations, garbage dump and farms to improve the independence of our home range estimate. Since the use of a reference smoothing factor (href) led to unrealistic estimates of home ranges (see Fig. S3 for details), we checked manually home range sizes using different smoothing parameters (h)^46^ and realistic estimates were found for h between 500–1000 m (see Fig. S4 for examples and more details on selection of smoothing parameters). We therefore chose to set the width around each point location to 750 m, using a constant kernel width for each individual allowing comparing ranging behaviour between individuals. To check for effects of temporal/spatial autocorrelation of locations on home range estimates, we recalculated 95%KDE using Brownian Bridge Movement Models (BBMM), which method specifically integrates movement paths in estimates of home range sizes^47^. However, both methods produced highly similar 95%KDE when using similar smoothing parameters, or estimates were highly correlated when using various custom made smoothing factors (details provided at Figs S4 and S5).

### Dominance data

Data on social dominance were collected in February 2016 (early breeding season) and August-September 2016 (post-breeding season). We noted all agonistic displacements between colour-ringed individuals around baits at the central feeding station (from a hide) between sunset and sunrise. In total, we observed 4593 displacements between 141 individuals that were involved in more than 20 displacements (average 65.1 ± 3.0 S.E., range 20–175), including 40 birds fitted with GPS loggers (19 males and 21 females: average 65.5 ± 6.4 S.E. displacements, range 20–175). Rank scores for each individual were determined using David’s score (the ‘compete’ package in R^48^, corrected for chance of encounter and thus independent of group size or visiting rate, see Suppl. Mat. Text S1 and Fig. S6 for details^49^. Scores obtained from the total dataset were used (details in Fig. S7). Rank was scaled between 0–1 with 1 being the most dominant bird.

### Statistical analyses

We conducted all statistical analyses using SAS 9.4 software (SAS Institute Inc., Cary, NC). We used a three-step approach to analyse our data. First, to quantify individual differences in the use of predictable and semi-predictable resources and movement behaviour we tested for repeatable individual differences in resource use (time spent at the main feeding station and farms, both square-root transformed), resource preference (ratio time spent at feeding station: farms, arctangent-transformed) and mobility (ranging behaviour (95KDE, log-transformed) and time spent flying (‘flight activity’, square-root transformed)). All transformed response variables were scaled by the standard deviation and mean-centred. Repeatability (R) was calculated based on a null model without main effects (i.e., non-adjusted), as the among-individual variance divided by the sum of the among-individual and the residual within-individual variance^50^, using univariate mixed-effect models with bird ID as a random effect and a Gaussian error distribution. To interpret factors influencing individual repeatability in behaviours, non-adjusted repeatability indices were compared with adjusted repeatability in full models including individual traits and environmental factors (see below). To test the significance of repeatability, we compared models with and without the random effect of bird ID using a likelihood ratio test LRT^51,52^.

Second, links between individual preferences for resources differing in predictability and mobility patterns were tested by analysing within- and between-individual correlations in monthly resource use, ranging behaviour and flight activity (based on average daily measurements) using bivariate mixed-effects models^50^. Significance of within-individual correlations was tested by comparing unconstrained models with models where the within-individual covariance was constrained to zero, again by applying an LRT test (to compare the χ^2^ against P(χ^2^, df = 1).

In the last part of our analyses, we tested the effects of individual attributes and territory location on resource preferences and mobility (dependent variables). First, we fitted LMMs with bird ID as random effect and included month, sex (male or female), dominance rank, age (in years) and territorial status (yes or no) as fixed effects. Since exploratory analyses showed that resource use and patterns of mobility varied strongly across the year in a non-linear manner (Fig. S2), we included month squared in all analyses. To test whether effects of dominance rank on resource use varied between the sexes and between territorial and non-territorial birds, we included two interactions: sex × rank and territorial status × rank in all models. We also included another two interactions (month squared × sex and month squared × dominance rank) to test for sex- or dominance-specific seasonal relationships. All covariates were mean and variance standardized. Finally, for territorial birds, we tested whether distance from the territory (nest location) to the central feeding station affected resource use and mobility, and whether territory location was explained by social rank (for males and females separately), using a LMM again with bird ID included as a random effect. Full models included all main fixed effects, random effects and interaction terms irrespective of their significance. Note that adjusted repeatability indices were calculated based on these full models.

### Ethic statements

Capture, banding and monitoring of Egyptian vultures were conducted under permits and following the protocols approved by the Cabildo Insular de Fuerteventura and the Dirección General de Protección de la Naturaleza (Viceconsejería de Medio Ambiente, Canarian Government) and following the protocols approved by the Ethic Committee of CSIC (CEBA-EBD-12-56), in accordance with the approved guidelines.

## Electronic supplementary material

Supplementary information
