# Supplementary material for: Food predictability and social status drive individual resource specializations in a territorial vulture
Source: Sci Rep. 2018 Oct 11;8:15155. doi: 10.1038/s41598-018-33564-y (PMC6181911; doi:10.1038/s41598-018-33564-y)
Supplement: Supplementary file 1 — Supplementary information [file 41598_2018_33564_MOESM1_ESM.docx]

**Supplementary information**

Food predictability and social status drive individual resource specializations in a territorial vulture

**Running header:** Resource **specialization** in vultures

**Authors:** Thijs van Overveld¹, Marina García-Alfonso¹, Niels J. Dingemanse², Willem Bouten³, Laura Gangoso¹^,^³, Manuel de la Riva¹, David Serrano¹, José A. Donázar¹

**
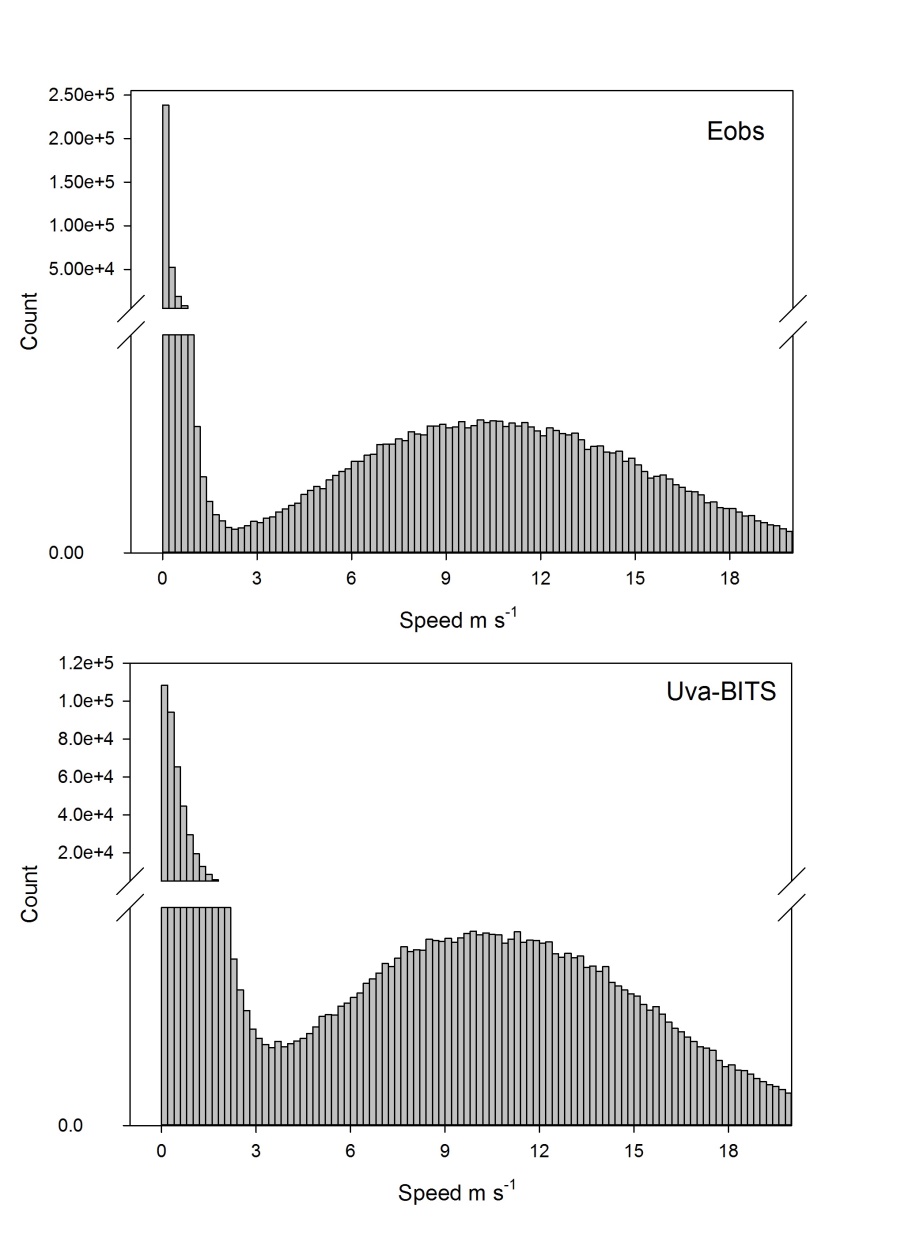
**

**Figure S1.** Frequency distribution of groundspeed measured by the two GPS-devices (Eobs and UvA-Bits). A threshold groundspeed of 3 m s^-1^ was used to distinguish between flight and non-flight behaviour. Running analyses with different thresholds for ground speed (2-5 m s^-1^ did not change the results, data not shown)


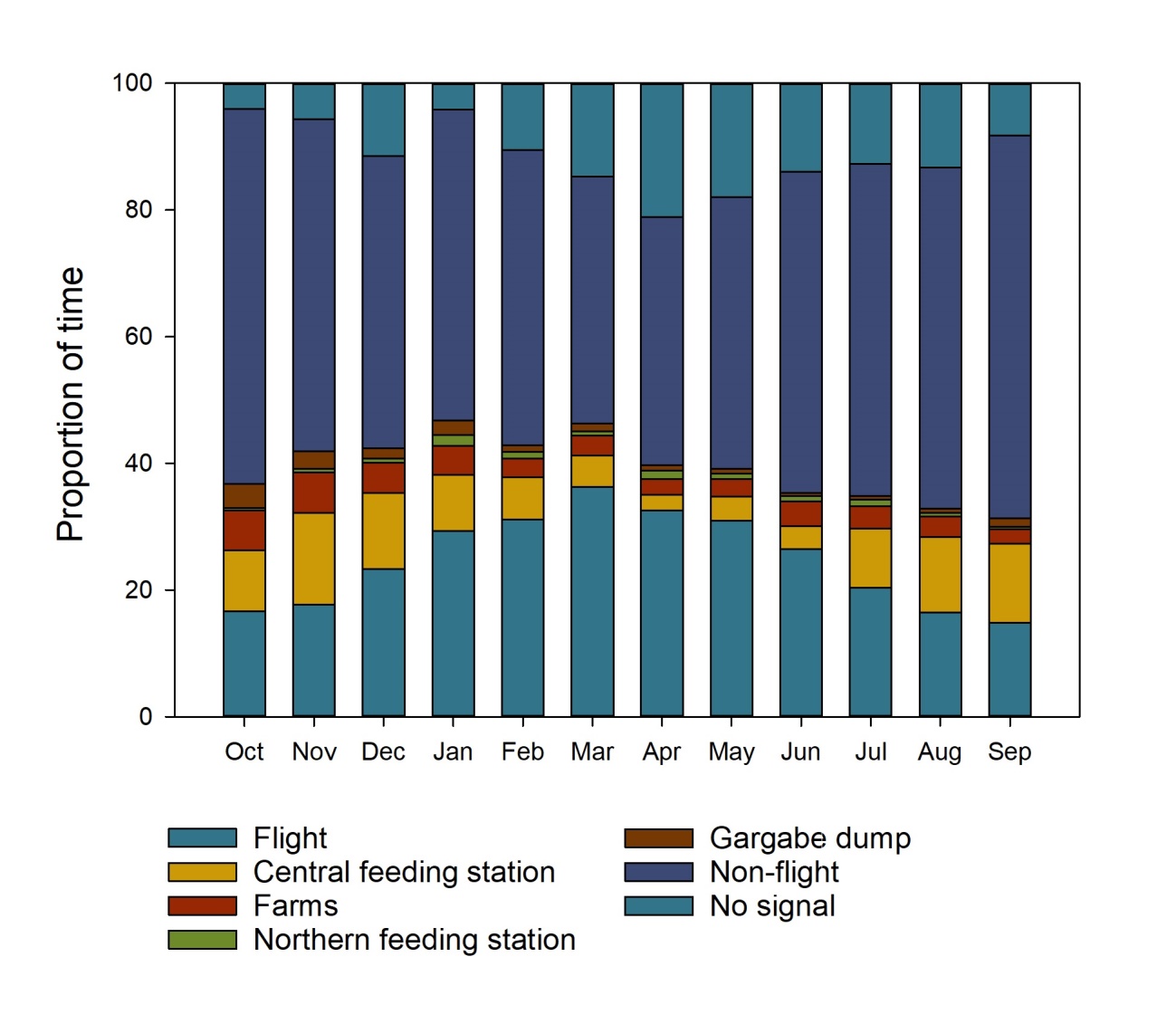
**Figure S2.** Average monthly time-budgets of GPS-logged Egyptian vultures (n = 45) on Fuerteventura between October 2015 and September 2016. Both the use of semi-predictable resources (farms) and predictable resources (feeding station) is strongly reduced during the breeding season (January-July). The high percentage of time with no signal between March and May is due to birds incubating eggs on nest in caves or on cliffs with bad reception. Note the clear preference for the central feeding station and to a lesser extent farms, while the feeding station in the north and the garbage dump are ignored by most birds. Also note that the remaining time occupied with non-flight behaviour mostly covers time spent resting, but may also include time spent consuming carcasses of wild animals (natural food resources).


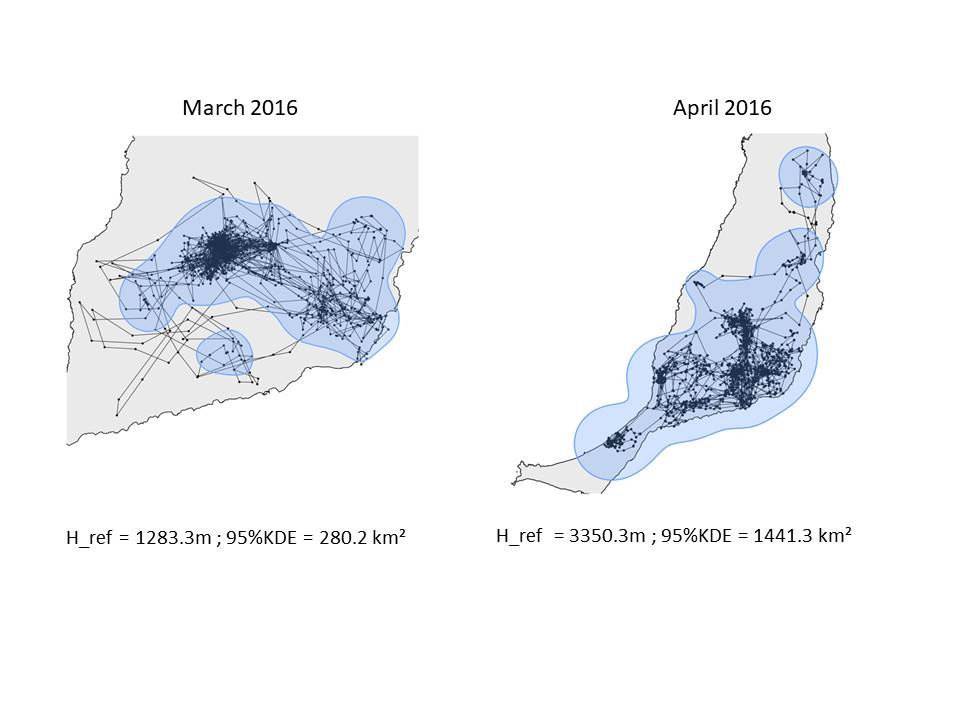


**Figure S3.** Examples showing over-smoothing of home-range sizes (device 6018, unpaired female) using a reference smoothing factor (H_ref). Default (reference) smoothing factor estimation is typically not reliable when ranging behaviours shows a multi-model distribution. Automatic calculation of a smoothing factor through the least-square cross validation algorithm did not work because of the high resolution of data resulting in small distances between GPS fixes.

**
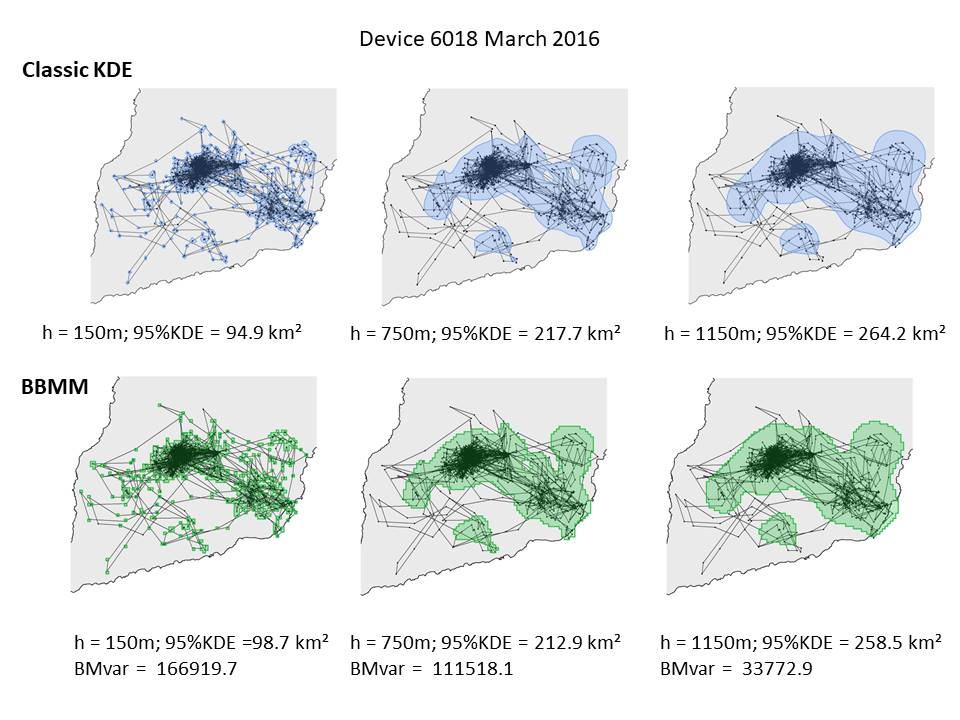

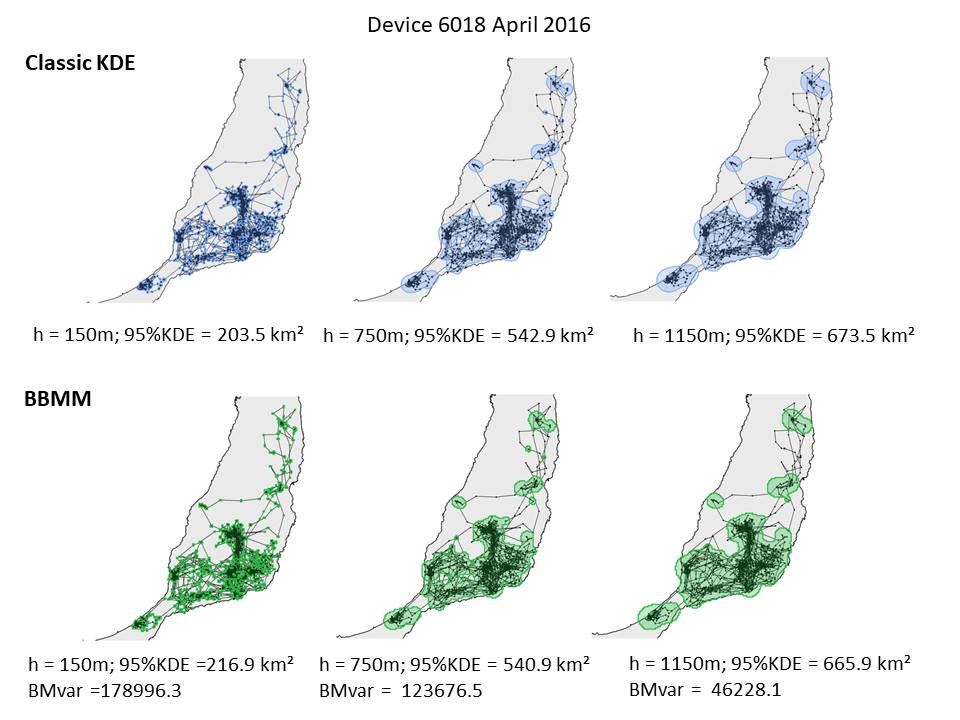
**

**Figure S4** Examples showing home-range sizes based on different manually chosen smoothing parameters, using fixed kernel contour method (‘classic method’, adehabitHR package, R version 3.03) and Brownian Bridge Movement Models (BBMM, with a maximum time lag of 60 min., using the BBMM package, R version 3.0). Both methods produced highly similar 95% kernel density estimates (95%KDE). While BBMM are intended to integrate time spent at locations and to take into account autocorrelations (by determining movements paths), time lag variation may cause little change to KDE measurements based on classic kernel contour methods when using data re-sampled at specific time-intervals (in our case 10 min.). In addition, Fuerteventura is a small island with little barriers. Movements paths of Egyptian vultures typically tend to largely overlap, since birds spend considerable time in certain areas (multimodal distribution of locations). Movements in-between these areas may be too fast (given relatively high flights speeds of Egyptian vultures) to become included in home range estimates.


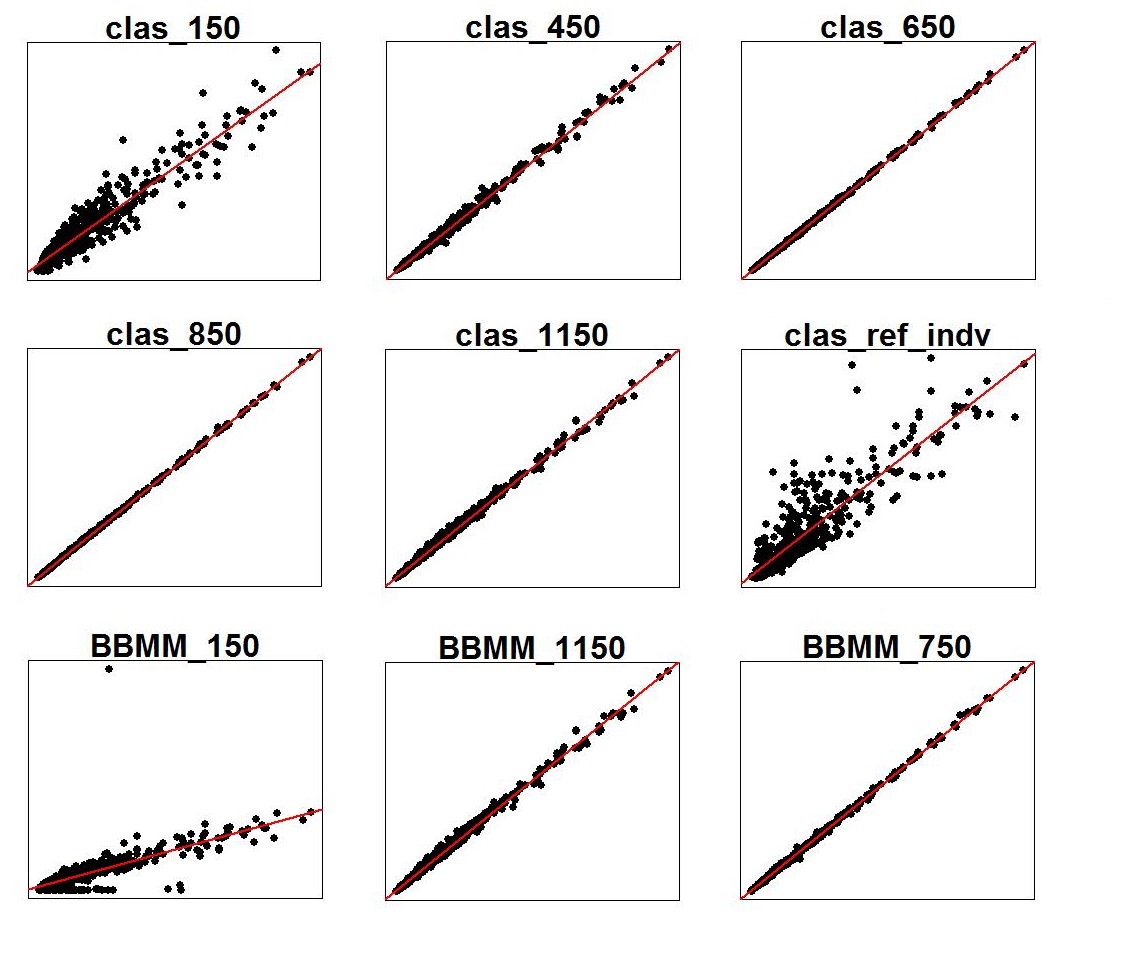


**Figure S5.**  Correlations between monthly home ranges ( 95%KDE) using the fixed kernel contour method (‘clas’, with h = 750, used in our analyses, on the x-axes), and 95%KDE calculated with different smoothing factors using a similar method and Brownian Bridge Movement Models (BBMM) (on the y-axes). Numbers in graph correspond the smoothing factor (h) in meters (href = 2528.9). All Pearson correlation coefficients > 0.86, p < .0001, n = 486 individual-months per graph.

**Figure S6.** Relationship between the number of displacements scored for each individual (n = 141, > 20 displacements) and the dominance score (David’s score), corrected for chance. A weak, but significant, positive correlation exists between the number of interactions and dominance score (Pearson correlation: r = 0.32, p = 0.001), which is due to dominant birds visiting the feeding station more frequently (see results).

**Figure S7**. Relationship between unstandardized David’s score calculated using either all interactions between 141 birds in the total datasets (4593 displacements) and scores calculated using interactions between GPS birds only (550 displacements) (Pearson correlation: r = 0.95, p <.0001)

**Text S1. The structure of dominance relations**

To examine whether the dominance relationships were linear we calculated the ‘adjusted linearity index h’ (de Vries 1995), using the ‘Compete’ package in R (Curley 2016). This index ranges from 0 (non-linear) to 1 (highly linear). To determine the statistical significance of h’, we tested whether this index differed significantly from the expected value for random dominance relations by running 10000 randomizations (corrected for unknown relationships by randomly filling in null dyads). Landau’s linearity indices h′ for both the total and GPS dataset were low (h′ = 0.07 and h′ = 0.14, respectively), although significant (p = 0.0001, p = 0.0017). The low h′ indexes are most likely due to the high number of null dyads in the datasets leading to a bias in these estimates (Shizuka and McDonald 2013). We therefore also calculated the directional consistency index (DCI) (van Hooff & Wensing 1987), which provides a measure for assessing how frequent a type of behaviour occurred in its more frequent direction relative to the total number of times it occurred (using the equation DCI = (H - L)/(H + L), where H is the total number of times the behaviour occurred in the direction of the higher frequency and L is the number of times in the less frequent direction). DCI scores were high in both datasets (DCI = 0.957 and DCI = 0.945 respectively), indicating a relatively strong linearity in hierarchy.

**Supporting References**

De Vries, H. (1995) An improved test of linearity in dominance hierarchies containing unknown or tied relationships *Animal Behaviour,* **50,** 1375-1389.

Curley, J.P. (2016) compete: Analyzing Social Hierarchies: R package version 0.1 -<https://github.com/jalapic/compete>.

Shizuka, D. & McDonald, D.B. (2013) A social network perspective on measurements of dominance hierarchies. *Animal Behaviour,* **83,** 925–934.

Van Hooff, J. & Wensing, J.A.B. (1987) *Dominance and its behavioural measures in a captive wolf pack*. Junk Publishers, Dordrecht, The Netherlands.

**Text S2. Monthly variation in resource use**

Average monthly flight activity was generally low during the non-breeding season (July-December) and substantially increased during the breeding season (January-June) (18.2% ± 7.0 SD, n = 250 and 31.1% ± 10.3, n = 236 respectively). Average monthly time spent at the central feeding station was lower during the breeding season compared to the non-breeding season (18.25h ± 23:02 SD, n = 236 and 42:16h ± 35:25 SD, n = 250, respectively). This difference was due to a lower (absolute) number of visits (14.4 ± 16.9 SD, n = 236 vs. 26.3 ± 21.1 SD, n = 250), shorter visiting bouts (01:15h ± 00:46 SD, n = 185 vs. 01:38h ± 00:46 SD, n = 226) and fewer individuals visiting this feeding site (30.8 ± 3.0 SD, n = 6 vs. 37.7 ± 1.8 SD, n = 6) during breeding. Foraging activities were also more dispersed during the breeding season compared to the non-breeding season (i.e., average monthly flight distance from central feeding station: 16.1 km ± 10.0 SD, n = 236 vs. and 11.8 km ± 9.7 SD, n = 250, respectively).

Average monthly time spent at farms was slightly lower during the breeding season compared to the non-breeding season (12:23h ± 14.29 SD, n = 236 and 15.54h ± 17:56 SD, n = 250, respectively). Visiting bouts at farms were shorter during the breeding season (00:59h ± 00:41 SD, n = 218 vs. 01:30h ± 01:14 SD, n = 242) while visiting rates were slightly higher (12.3 ± 11.8SE, n = 236 vs. 10.6 ± 10.1SD, n = 250, respectively). Farms visited during the breeding season were located further away from the central feeding station compared to the farms visited during the non-breeding season (average monthly distance: 16.7km ± 11.3 SD, n = 222 and 11.8km ± 9.8 SD, n = 246, respectively). The average monthly number of farms visited (per individual) did not differ between the breeding and non-breeding season (4.8 ± 4.0 SD, n = 236 and 4.6 ± 2.9 SD, n = 250 respectively).

|  |  | Territorial females | |  |  | Territorial males | |  |  | Non-territorial birds | |  |
| --- | --- | --- | --- | --- | --- | --- | --- | --- | --- | --- | --- | --- |
|  |  |  |  |  |  |  |  |  |  |  |  |  |
| dependent | fixed effects | *β* (SE) | F_NUMdf, DENdf_ | *P* |  | *β* (SE) | F_NUMdf, DENdf_ | *P* |  | *β* (SE) | F_NUMdf, DENdf_ | *P* |
| variable |  |  |  |  |  |  |  |  |  |  |  |  |
|  |  |  |  |  |  |  |  |  |  |  |  |  |
| *Time at central feeding station* | |  |  |  |  |  |  |  |  |  |  |  |
|  | Sex | n/a | n/a | n/a |  | n/a | n/a | n/a |  | 0.23 (0.37) | 0.39 _1,24.5_ | 0.54 |
|  | Dominance rank | 0.82 (0.23) | 12.49 _1,16.8_ | **0.0026** |  | -0.02 (0.46) | 0.00 _1,7.83_ | 0.97 |  | n/a | n/a | n/a |
|  | Distance territory | -0.60 (0.18) | 11.10 _1,18.1_ | **0.0037*** |  | -0.35 (0.16) | 4.77 _1,8.42_ | 0.06 |  | n/a | n/a | n/a |
|  | Month | -0.10 (0.05) | 3.55 _1,128_ | 0.06 |  | 0.02 (0.06) | 0.09 _1,89_ | 0.77 |  | -0.02 (0.05) | 0.11 _1,191_ | 0.74 |
|  | Month² | 0.93 (0.11) | 75.95 _1,127_ | **<.0001** |  | 0.25 (0.11) | 5.22 _1,88.1_ | **0.0247** |  | 0.27 (0.07) | 17.66 _1,189_ | **<.0001** |
|  | Month²*Dominance rank | -0.26 (0.09) | 7.74 _1,128_ | **0.0062** |  | -0.60 (0.16) | 13.14 _1,88.1_ | **0.0005** |  | n/a | n/a | n/a |
|  | Month²*Distance territory | 0.20 (0.07) | 8.30 _1,140_ | **0.0046*** |  | 0.22 (0.06) | 14.07 _1,88.1_ | **0.0003** |  | n/a | n/a | n/a |
|  | Month²*sex | n/a | n/a | n/a |  | n/a | n/a | n/a |  | 0.06 (0.13) | 0.19 _1,189_ | 0.66 |
|  | * Significant when entered in the model without dominance rank | | | |  |  |  |  |  |  |  |  |
| *Time at farms* |  |  |  |  |  |  |  |  |  |  |  |  |
|  | Sex | n/a | n/a | n/a |  | n/a | n/a | n/a |  | -0.32 (0.27) | 1.35 _1,33.8_ | 0.25 |
|  | Dominance rank | -0.13 (0.20) | 0.46_1,11.3_ | 0.51 |  | 1.51 (0.53) | 7.98 _1,6.48_ | **0.0278** |  | n/a | n/a | n/a |
|  | Month | -0.23 (0.05) | 18.61 _1,130_ | **<.0001** |  | -0.19 (0.06) | 10.16 _1,90.7_ | **0.0020** |  | -0.20 (0.05) | 13.76 _1,194_ | **0.0003** |
|  | Month² | 0.16 (0.07) | 5.93 _1,129_ | **0.0163** |  | 0.24 (0.07) | 10.54 _1,89.6_ | **0.0016** |  | -0.04 (0.10) | 2.90 _1,192_ | 0.09 |
|  | Month²*Dominance rank | 0.09 (0.10) | 0.79 _1,128_ | 0.38 |  | 0.21 (0.18) | 1.76 _1,88.5_ | 0.19 |  | n/a | n/a | n/a |
|  | Month²*sex | n/a | n/a | n/a |  | n/a | n/a | n/a |  | -0.06 (0.29) | 1.32 _1,192_ | 0.22 |
|  |  |  |  |  |  |  |  |  |  |  |  |  |
| *Home range size* |  |  |  |  |  |  |  |  |  |  |  |  |
|  | Sex | n/a | n/a | n/a |  | n/a | n/a | n/a |  | -0.74 (0.20) | 14.21 _1,18.5_ | **0.0014** |
|  | Dominance rank | 0.16 (0.26) | 0.38 _1,14.5_ | 0.55 |  | 0.37 (0.54) | 0.47 _1,10.3_ | 0.51 |  | n/a | n/a | n/a |
|  | Month | -0.13 (0.04) | 8.86 _1,128_ | **0.0035** |  | -0.31 (0.08) | 14.51 _1,90.9_ | **0.0003** |  | -0.01 (0.05) | 0.08 _1,196_ | 0.77 |
|  | Month² | 0.56 (0.10) | 33.46 _1,127_ | **<.0001** |  | 0.13 (0.15) | 0.65 _1,89.1_ | 0.42 |  | -0.50 (0.06) | 68.4 _1,193_ | **<.0001** |
|  | Month²*Dominance rank | -0.38 (0.08) | 20.09 _1,127_ | **<.0001** |  | -0.47 (0.24) | 3.88 _1,89_ | **0.0520** |  | n/a | n/a | n/a |
|  | Month²*sex | n/a | n/a | n/a |  | n/a | n/a | n/a |  | 0.19 (0.12) | 2.45 _1,192_ | 0.12 |
|  |  |  |  |  |  |  |  |  |  |  |  |  |
| *Flight activity* |  |  |  |  |  |  |  |  |  |  |  |  |
|  | Sex | n/a | n/a | n/a |  | n/a | n/a | n/a |  | -0.11 (0.22) | 0.29 _1,17.9_ | 0.59 |
|  | Dominance rank | 0.46 (0.26) | 3.18 _1,16.4_ | 0.09 |  | 0.45 (0.36) | 1.61 _1,13.2_ | 0.23 |  | n/a | n/a | n/a |
|  | Month | 0.10 (0.06) | 2.67 _1,127_ | 0.10 |  | 0.23 (0.07) | 13.01 _1,91.8_ | **0.0005** |  | 0.01 (0.04) | 5.59 _1,193_ | **0.0191** |
|  | Month² | -0.58 (0.13) | 18.75 _1,126_ | **<.0001** |  | -1.05 (0.12) | 71.67 _1,89.4_ | **<.0001** |  | -0.78 (0.05) | 252.25 _1,191_ | **<.0001** |
|  | Month²*Dominance rank | -0.19 (0.11) | 2.55 _1,127_ | 0.11 |  | -0.32 (0.19) | 2.86 _1,89.3_ | 0.09 |  | n/a | n/a | n/a |
|  | Month²*sex | n/a | n/a | n/a |  | n/a | n/a | n/a |  |  | 4.64 _1,190_ | **0.0326** |
|  |  |  |  |  |  |  |  |  |  |  |  |  |
|  |  |  |  |  |  |  |  |  |  |  |  |  |
| *Total number of farms* | |  |  |  |  |  |  |  |  |  |  |  |
|  | Sex | n/a | n/a | n/a |  | n/a | n/a | n/a |  | -0.67 (0.25) | 7.00 _1,39.6_ | **0.0116** |
|  | Dominance rank | -0.84 (0.32) | 6.50 _1,15.9_ | **0.0215** |  | 0.77 (0.49) | 2.45 _1,9.45_ | 0.15 |  | n/a | n/a | n/a |
|  | Month | -0.02 (0.07) | 0.09 _1,128_ | 0.77 |  | -0.20 (0.08) | 6.91 _1,90.4_ | **0.0101** |  | -0.27 (0.06) | 23.23 _1,196_ | **<.0001** |
|  | Month² | -0.22 (0.14) | 2.32 _1,127_ | 0.13 |  | 0.23 (0.14) | 2.68 _1,88.4_ | 0.11 |  | -0.21 (0.07) | 8.75 _1,193_ | **0.0035** |
|  | Month²*Dominance rank | 0.32 (0.12) | 6.93 _1,128_ | **0.0095** |  | -0.07 (0.22) | 0.10 _1,88.3_ | 0.75 |  | n/a | n/a | n/a |
|  | Month²*sex | n/a | n/a | n/a |  | n/a | n/a | n/a |  |  | 0.11 _1,193_ | 0.74 |
|  |  |  |  |  |  |  |  |  |  |  |  |  |

**Table S.1** Results of GLMM on seasonal effects and individual attributes affecting monthly time spent at the central feeding station (square-root transformed), time spent at farms (square-root transformed), home range size (logKDE95), flight acivity (time spent flying, square-root transformed) and number of farms visited (square-root transformed) for territorial males (9 individuals, 101 months), territorial females (13 individuals, 142 months) and non-territorial birds (18 individuals (10 males and 8 females), 209 months). All transformed response variables were scaled by the standard deviation and mean-centred.
